# Supplementary material for: Relationship between IgA Nephropathy and Porphyromonas gingivalis; Red Complex of Periodontopathic Bacterial Species
Source: Int J Mol Sci. 2021 Dec 1;22(23):13022. doi: 10.3390/ijms222313022 (PMC8657970; doi:10.3390/ijms222313022)
Supplement: Supplementary file 1 [file ijms-22-13022-s001.zip › ijms-1413947-supplementary.pdf]

Supplemental Table S1: Summary of Body Weight Chage

		Numbers	Average	SD	
14days	Control	10	27.1	2.1	
	Pg	10	26.9	2.9	NS
28days	Control	10	28.4	3.7	
	Pg	10	25.6	4.2	NS
42days	Control	10	29.4	2.0	
	Pg	10	28.6	2.7	NS
56days	Control	10	30.8	2.8	
	Pg	12	30.0	2.6	NS

Supplemental Table S2　Summary of Serum Blood Urea Nitrogen

		Numbers	Average	SD	
14days	Control	10	18.2	3.7	
	Pg	10	18.2	3.5	NS
42days	Control	10	20.8	2.0	
	Pg	10	17.9	3.5	NS
56days	Control	10	18.6	2.6	
	Pg	12	18.1	2.0	NS

Supplemental Table S3 Summary of Albuminuria

		Numbers	Average	SD	
14days	Control	4	0.06	0.03	
	Pg	5	0.05	0.02	NS
42days	Control	2	0.05	0.02	
	Pg	2	0.09	0.07	NS
56days	Control	5	0.26	0.11	
	Pg	3	0.22	0.04	NS
